# Growth differentiation factor 15 predicts physical function impairment in Spanish older adults: a real-world prospective study

**DOI:** 10.1007/s11357-025-01779-3

**Published:** 2025-07-02

**Authors:** Karine Ferreira de Campos, Esther García-Esquinas, Antonio Buño-Soto, Mercedes Sotos-Prieto, Adrián Carballo-Casla, Fernando Rodríguez-Artalejo, Rosario Ortolá

**Affiliations:** 1 Department of Preventive Medicine and Public Health, School of Medicine, Universidad Autónoma de Madrid, Calle del Arzobispo Morcillo 4, 28029 Madrid, Spain; 2 CIBER of Epidemiology and Public Health (CIBERESP), Madrid, Spain; 3 National Center for Epidemiology, Carlos III Health Institute, Madrid, Spain; 4 Department of Laboratory Medicine, La Paz University Hospital-IdiPaz, Madrid, Spain; 5 Department of Environmental Health and Nutrition, Harvard T.H. Chan School of Public Health, Boston, MA USA; 6 IMDEA Food Institute. CEI UAM+CSIC, Madrid, Spain; 7 Aging Research Center, Department of Neurobiology, Care Sciences and Society, Karolinska Institute & Stockholm University, Stockholm, Sweden

**Keywords:** GDF15, SPPB, Frailty, Agility, Mobility

## Abstract

Growth differentiation factor 15 (GDF15) is an emerging biomarker of disease burden, but few studies explore its association with physical function impairment using validated objective measures. We evaluated the cross-sectional and longitudinal association between GDF15 and impaired physical function in older adults, assessed with five validated tools. Data were collected from 2481 individuals ≥ 65 years-old in the Seniors-ENRICA-2 cohort. Serum GDF15 was measured once, at baseline, and physical function was assessed at baseline and after 2.2 years using the following measures: lower-extremity performance (Short Physical Performance Battery), agility (Nagi scale), mobility (Nagi and Rosow & Breslau questionnaires), weakness (measured grip strength) and frailty (Deficit Accumulation Index). Analyses were performed with logistic regression and adjusted for relevant potential confounders.The odds ratios (95% confidence interval) per 25% increment in GDF15 in cross-sectional and prospective analyses were: 1.06 (1.01–1.12) and 1.18 (1.08–1.30) for reduced lower-extremity performance; 1.18 (1.12–1.26) and 1.21 (1.08–1.35) for impaired agility; 1.19 (1.11–1.27) and 1.21 (1.08–1.35) for impaired mobility; 1.15 (1.09–1.22) and 1.13 (1.02–1.25) for weakness; and 1.24 (1.16–1.33) and 1.18 (1.06–1.31) for frailty. Results were similar among participants without cardiovascular disease or diabetes. Higher levels of serum GDF15 are associated with prevalent and incident impaired physical function in older adults. GDF15 could serve as a convenient biomarker to identify older adults at risk of functional decline. Further studies should elucidate the underlying mechanisms, explore its potential to improve risk prediction, and assess the therapeutic value of modifying GDF15.

**Supplementary Information:**

The online version contains supplementary material available at 10.1007/s11357-025-01779-3.

## Introduction

Aging is associated with gradual changes in body composition, including the loss of up to 40% of skeletal muscle mass and strength from the second to the eighth decade of life [1]. Low skeletal muscle mass is associated with lower extremity physical performance, functional impairment, and frailty [2, 3]. Frail individuals often experience a significant decline in their physical and functional capacities, and have an increased risk of developing disabilities, especially in community-dwelling settings [4–6]. Over 1 billion people worldwide experience some form of disability, and this number will increase drastically due to a rise in chronic health conditions resulting from population ageing [7]. In Spain, the estimated prevalence of some forms of disability among older adults is 73%, with 59% presenting at least mild impairment in agility, 52% in mobility, and 19% in daily living activities [8]. However, while disability partly results from specific diseases, its pathogenesis involves complex, multifactorial processes that are not fully elucidated and may not be detected through clinical measures alone. Biomarkers may help uncover early biological changes underlying disability, enabling the identification of individuals at risk of functional decline.

Growth differentiation factor 15 (GDF15) is a member of the transforming growth factor-β superfamily, which, under normal conditions, is expressed in low amounts in several tissues, such as liver, intestine, and kidneys [9, 10]. Upregulation of GDF15 occurs as a response to mitochondrial, metabolic, and inflammatory stress. Since elevated levels of GDF15 have been implicated in the development, progression, or prognosis of several chronic diseases [11–18], it is an emerging biomarker of disease burden in chronic conditions, including cardiac and renal failure, chronic liver disease, diabetes, obesity, various cancers, chronic inflammatory diseases, and mitochondrial diseases [19–22]. GDF15 is also a strong predictor of all-cause death, independently of other risk factors and mortality biomarkers [23], supporting its role as a good biomarker of the aging process and age-related chronic diseases in older people. Additionally, there is recent evidence that GDF15 predicts physical function decline in hypertensive older adults [24] and it has been associated with lower-limb physical performance [25], muscle weakness, cachexia and decreased cognitive function [25–29] in separate analyses. Therefore, we have focused on GDF15 because it is increasingly recognized as a pleiotropic marker of biological aging and multisystem stress. Beyond its association with mortality and chronic disease, GDF15 has been mechanistically linked to appetite regulation, mitochondrial dysfunction, and catabolic states, including cachexia and muscle wasting [27–31]. These processes are known to contribute to physical function loss in older adults, suggesting a plausible role for GDF15 as an upstream signal of functional decline.

However, to our knowledge, there are few prospective studies specifically aiming to assess the relation between GDF15 and the risk of impaired physical function combining multiple validated measures. Therefore, we aimed to assess whether serum GDF15 levels independently predict physical impairment in older adults in multiple domains, using validated functional outcomes, and to assess the potential translational relevance of this biomarker for risk stratification in geriatric populations. We hypothesize that GDF15 precedes functional decline by reflecting early physiological dysregulation that predisposes to loss of function, independently of age and comorbidity, based on prior evidence linking elevated GDF15 to mitochondrial dysfunction, anorexia, and muscle wasting—key drivers of reduced physical performance.

## Methods

### Study population and design

We used data from the Seniors-ENRICA-2 cohort [32], which was set up in 2015–2017. In total, 3273 individuals were selected by stratified random sampling of community-dwelling individuals aged ≥ 65 years holding a national healthcare card and residing in the Madrid region (Spain). The study included a computer-assisted telephone interview to collect information on sociodemographic characteristics, lifestyle, and morbidity. Also, two home visits were subsequently conducted by trained staff to assess diet and physical performance, as well as to collect blood samples [32]. In 2019, participants were re-interviewed, and a new physical exam was performed [33]. All participants provided informed written consent, and the study was approved by the Clinical Research Ethics Committee of the “La Paz” University Hospital in Madrid (Protocol #HULP-PI 1793).

### Study variables

#### Serum GDF15

Serum GDF15 was measured only at baseline. Twelve-hour fasting blood samples were collected from each participant at the first home visit in rapid serum tubes with thrombin-based clot activator and polymer gel (Becton Dickinson) [19]. Within 1 h after collection, tubes were centrifuged at 2520 g and room temperature (20–23 ºC) for 10 min, and serum was aliquoted, frozen at − 80 ◦C, and stored up to 3.6 years at the Department of Preventive Medicine and Public Health of the Universidad Autónoma de Madrid. Serum GDF15 was measured at the Department of Laboratory Medicine of “La Paz” University Hospital by an electrochemiluminescence Elecsys® immunoassay method using a cobas® 6000 analyzer (Roche Diagnostics). This assay is based on the sandwich immunoassay principle, utilizing biotin-streptavidin technology for enhanced specificity and sensitivity. Samples of 35 μL were first incubated for 9 min with a biotinylated murine monoclonal anti-GDF15 antibody labeled with a ruthenium complex [Tris(2,2′-bipyridyl)ruthenium(II)], forming a sandwich complex when GDF15 is present. Streptavidin-coated microparticles were added in a second 9-min incubation period. Unbound substances were then removed using a ProCell washing solution (Roche Diagnostics), and the resulting antigen–antibody complexes were detected via electrochemiluminescence using the cobas® analyzer. Results were determined through an instrument-specific calibration curve, generated by 2-point calibration and a master curve provided via the reagent barcode.

Controls were run in quadruplicate each day. The intra-assay coefficient of variation was 2.8% for a mean concentration of 7332 pg/mL, and 3.1% for a mean concentration of 1422 pg/mL, and the inter-assay coefficient of variation was 5.4% for a mean concentration of 7343 pg/mL, and 7.7% for a mean concentration of 1428 pg/mL. These values are comparable to those obtained previously: 0.7% to 7.7% and 1.7% to 8.6% for within-run and within-laboratory precision, respectively, for samples containing 670–16039 ng/L [34]. The assay also shows strong correlation and agreement with manual immunoradiometric assays (IRMA) and ELISA [34]. Samples with GDF15 concentrations above the measuring range (≥ 3500 pg/mL) were diluted with Diluent MultiAssay, and those below the level of detection or quantitation (< 400 pg/mL, n = 2) were estimated by dividing the measured value by the square root of 2, a method commonly used in biomarker research to handle low-concentration data, ensuring that undetectable values are incorporated into analyses without introducing excessive bias.

#### Physical function

##### Reduced lower extremity performance

It was assessed at baseline and at the 2.2-year follow-up with the Short Physical Performance Battery (SPPB), which included three tests: 1) Balance test, 2) Gait- speed test and 3) Sit-to-stand test [35]. 1) Balance testing included a side-by-side, a semi tandem and a tandem stand: Participants were first asked to stand with their feet together. Those who were able to stand for 10 s in this position were then tested in the semi tandem position, where the heel of one foot is placed to the side of the big toe of the other foot. Finally, those who were able to stand for 10 s in the semi tandem were tested in the full-tandem stand, where the heel of one foot was placed in front of the toes of the other foot. A score of 0 in the balance test indicates the inability to stand in any of the positions; a score of 4 indicates a full-tandem stand for 10 s [35]. 2) Gait speed was estimated as the time taken to walk 3 m. In this test, 0 points indicated that the test was not performed, while scores of 1, 2, 3, or 4 were assigned to participants who completed the walk in > 5.6, > 4–5.6, > 3.1–4 and ≤ 3.1 s, respectively [35]. 3) The sit-to stand test consisted of standing up and sitting down from a chair five times repeatedly, with arms crossed across the chest. A score of 0 was given if a participant was unable to perform the five chair stands, while scores of 1, 2, 3, or 4 were assigned to participants who completed five chair stands in ≥ 16.7, 13.7 to < 16.7, 11.2 to < 13.7 and < 11.2 s, respectively [35]. The total SPPB score was calculated as the sum of the three components, with a range from 0 (worst) to 12 (best performance). Participants with a score ≤ 9 were deemed to have reduced performance of the lower extremity. The SPPB score threshold of 9 is a clinical cut-off commonly used to identify reduced lower extremity performance in older adults across various studies (33, 36–39). For the SPPB components, a task impairment was defined if there was a score ≤ 3 for each test [35].

##### Agility and mobility impairment

Agility and mobility were assessed at baseline and at the 2.2-year follow-up using brief self-reported questionnaires designed to capture perceived difficulties with movement and balance in daily life. Although these measures are less granular than performance-based tests, they provide complementary information by reflecting subjective experience and early signs of functional limitation. Agility was assessed to evaluate the participant’s self-reported ability to perform movements involving flexibility, coordination, and lower-body strength. Although correlated with general mobility, agility represents a distinct functional domain: mobility is about movement capability, while agility is about movement efficiency and adaptability.

Impaired agility was defined when study participants answered “a lot” to the following question from the Nagi scale [40]: “On an average day, would your current health limit you in bending and kneeling?” The categories of response were “yes, a lot,” “yes, a little, and “not at all.”

Likewise, patients were considered to have mobility impairment if answering “a lot” to any of the following questions from the Nagi and Rosow and Breslau scales [40, 41]: “On an average day with your current health, would you be limited in the following activities: (1) lifting or carrying a shopping bag?; (2) climbing one flight of stairs?; (3) walking several city blocks (a few hundred meters)?”.

##### Weakness

Grip strength was measured at baseline and at the 2.2-year follow-up with a Jamar dynamometer (highest of 2 consecutive measurements in the dominant hand). Participants in the cohort-specific lowest quintile, adjusted for sex and body mass index (BMI), were considered to have weakness, in accordance with other large cohort studies [42–44].

##### Frailty

Frailty was evaluated at baseline and at the 2.2-year follow-up using a Deficit Accumulation Index (DAI) based on the Rockwood’s multidimensional frailty index [45, 46]. We developed a 52-item DAI in our cohort comprising the following four domains: functional, self-rated health/vitality, mental health, and morbidity/health services use. The DAI was defined as the proportion of health deficits present in an individual out of the total number of 52 deficits considered, ranging from 0 (lowest) to 100% (highest deficit accumulation) [47]. Participants in the cohort-specific higher quintile of the DAI score were considered frail, since in other large cohort studies assessing frailty in older adults using a deficit accumulation approach, those in the highest quintile were at higher risk [48, 49].

#### Other variables

Information was collected at baseline on potential confounders of the study associations, which included the following sociodemographic and lifestyle variables: age, sex, educational level (primary or less, secondary, or university), smoking status (never, former, or current smoker), alcohol consumption [never, former, moderate (≤ 10 g/d in women and ≤ 20 g/d in men), or heavy drinker], leisure-time physical activity (metabolic equivalents of task-hour/week) [32], sedentary behavior (time spent watching television) [32], diet quality (measured with the 14-point Mediterranean diet adherence screener (MEDAS) [50]), sleep time (hours/day), and energy intake (kcal/day). The following clinical variables were also collected: body mass index (BMI), systolic blood pressure (SBP), fasting serum glucose, creatinine, and LDL cholesterol (LDL-c), as well as history of cardiovascular disease (CVD) and diabetes. Energy intake was derived from habitual diet information obtained from the validated diet history using appropriate food composition tables [51], and BMI was calculated as weight in kilograms divided by squared height in meters, both measured in standardized conditions [52]. SBP was measured 3 times under standardized conditions using the validated device Mobil-O-Graph and the average of the second and third measurements was used for analyses. Fasting serum glucose and creatinine were measured with colorimetric enzymatic methods using Atellica® solution (Siemens Healthineers); and LDL-c was calculated with the Friedewald formula (LDL = total cholesterol – triglycerides/5 – HDL). Last, CVD was ascertained by asking the study participants if they had been previously diagnosed with myocardial infarction, stroke, or heart failure; and diabetes if they reported a diagnosis of diabetes, had been prescribed antidiabetic medication, or had a fasting blood glucose ≥ 126 mg/dL.

### Statistical analyses

From the initial sample of 3273 participants, we excluded 684 due to missing GDF15 measurements, and then 108 for lacking data on potential confounders. Thus, a total of 2481 participants were included in the cross-sectional analyses (eFig. 1). For the prospective analyses, after excluding participants due to death, loss to follow-up, and lack of data on all the five variables of interest, the analytical samples comprised: 1120 older adults with no reduced lower-extremity performance, 1279 without impaired agility, 1430 without impaired mobility, 1217 without weakness, and 1480 without frailty at baseline (eFig. 2).

The differences in the distribution of GDF15 by sociodemographic, lifestyle and clinical characteristics of study participants were the first to be tested. Next, the cross-sectional and prospective associations between concentrations of GDF15 and reduced lower-extremity performance, impaired agility and mobility, weakness and frailty were summarized with odds ratios (OR) and their 95% confidence interval (CI) obtained from logistic regression models. Physical function outcomes were primary modeled as binary variables (‘impaired’ vs. ‘non-impaired’) using clinically validated thresholds adopted in geriatric research (SPPB ≤ 9 for reduced performance, lowest sex- and BMI-specific quintile for grip strength, highest frailty index quintile for frailty) [33, 36–44, 48, 49]. This approach facilitates interpretability in clinical and public health settings, and comparability with prior studies. To assess robustness, we also conducted supplementary analyses, modeling lower-extremity performance, weakness, and frailty as continuous variables.

GDF15 concentrations were modeled as 1) log-transformed continuous variable (per 25% increment); to normalize the positively skewed distribution of GDF15 levels and improve model fit, 2) quartiles; to explore dose–response patterns and identify potential non-linear trends; and 3) restricted cubic splines with knots at the 10th, 50th and 90th percentiles to evaluate potential nonlinear relationships without assuming linearity. Log-transformation of GDF15 has been employed in several previous studies examining its associations with health outcomes in aging populations [11, 16, 53, 54]. Also, interpretation of associations per 25% relative increase is more biologically meaningful than per unit changes. Important confounders were included in models as follows: model 1 adjusted for age, sex, and educational level; model 2 further adjusted for smoking status, alcohol consumption, physical activity, time watching TV, energy intake, sleep time and diet quality (MEDAS score); and model 3 further adjusted for BMI, SBP, serum glucose, serum creatinine, LDL-c, CVD, and diabetes.

To assess the robustness of our results and minimize reverse causation, we replicated the analyses with additional adjustment for depression requiring treatment, cognitive deterioration (defined as a Mini-Mental State Examination score < 24), appetite (very poor, poor, rather good, very good) or levels of interleukin-6 (IL-6) at baseline, as well as excluding participants with CVD or diabetes. Finally, we assessed whether sex or age modified the study associations by testing interaction terms defined as the product of GDF15 by categories of such variables. Since no significant interactions were found, results are presented for the total sample.

Statistical significance was set at a 2-sided P value < 0.05. Analyses were performed with Stata®, version 15 (StataCorp).

## Results

The geometric mean [geometric standard deviation] for serum levels of GDF15 was 1242 pg/mL [1.64] (Table 1). GDF15 increased with age and was higher among men, current smokers, former drinkers, and participants who had a poorer diet, lower physical activity, more than 8-h sleep, higher BMI, higher glucose and creatinine levels, lower LDL-c levels, and those with diabetes and CVD (Table 1).

**Table 1:** GDF15 concentrations by baseline characteristics of study participants

	**n (%)**	**Geometric mean (GSD)**
**Overall**^**a**^	2481 (100)	1242 (1.64)
**Age, years**		
65 to < 70	1134 (46)	1107 (1.60)*
70 to 93	1347 (54)	1368 (1.63)
**Sex**		
Men	1173 (47)	1313 (1.65)*
Women	1308 (53)	1181 (1.62)
**Education**		
Primary or less	1573 (63)	1258 (1.63)
Secondary	467 (19)	1199 (1.67)
University	441 (18)	1231 (1.64)
**Smoking status**		
Never smoker	1306 (53)	1198 (1.61)*
Former smoker	944 (38)	1256 (1.66)
Current smoker	231 (9)	1449 (1.65)
**Alcohol consumption**		
Never drinker	469 (19)	1307 (1.71)*
Moderate drinker	1314 (53)	1231 (1.62)
Heavy drinker	545 (22)	1176 (1.6)
Former drinker	153 (6)	1390 (1.68)
**MEDAS score **^**∫**^		
< 7	849 (34)	1278 (1.67)*
7 to 8	1097 (44)	1237 (1.63)
> 8	535 (22)	1195 (1.61)
**Physical activity (METs-h/week)**^**¥**^		
1 st tertile	952 (38)	1336 (1.70)*
2nd tertile	715 (29)	1215 (1.60)
3rd tertile	814 (33)	1161 (1.58)
**Time watching TV (hours/day)**		
< 3	896 (36)	1221 (1.63)
3 to < 4	715 (29)	1227 (1.65)
≥ 4	870 (35)	1275 (1.64)
**Sleep time at night (hours)**		
< 7	966 (39)	1254 (1.67)*
7 to 8	1317 (53)	1205 (1.60)
> 8	198 (8)	1438 (1.71)
**BMI (kg/m**^**2**^**)**		
< 25	667 (27)	1213 (1.68)*
≥ 25 to < 30	1170 (47)	1216 (1.61)
≥ 30	644 (26)	1320 (1.64)
**SBP (mmHg)**		
< 130	1022 (41)	1223 (1.65)
≥ 130	1459 (59)	1255 (1.63)
**Glucose (mg/dL)**		
< 100	1566 (63)	1124 (1.54)*
≥ 100	915 (37)	1473 (1.73)
**LDL-c (mg/dL)**		
< 130	1773 (71)	1315 (1.67)*
≥ 130	708 (29)	1076 (1.50)
**Energy intake (kcal/day)**^**ǂ**^		
1 st tertile	827 (33)	1258 (1.70)
2nd tertile	827 (33)	1220 (1.60)
3rd tertile	827 (33)	1247 (1.61)
**Creatinine (mg/dL)**		
≤ 1.2	2393 (96)	1212 (1.61)*
> 1.2	88 (4)	2406 (1.68)
**CVD**		
No	2397 (97)	1230 (1.63)*
Yes	84 (3)	1625 (1.75)
**Diabetes Mellitus**		
No	1983 (80)	1128 (1.52)*
Yes	498 (20)	1821 (1.78)

*a.* Participants that had at least 1 measure for SPPB, agility, mobility, grip strength or DAI score

^*^p < 0.05 for differences among groups, analyzed with t tests or one-way analysis of variance, as appropriate

^**∫**^ MEDAS score (Mediterranean Diet Adherence Screener, range: 0 to 14). ^**¥**^ Physical activity, tertiles: first: ≤ 17.5; second: > 17.5 to ≤ 33.5; third: > 33.5 METs-h/week. ^**ǂ**^ Energy intake, tertiles: first: ≤ 1802; second: > 1802 to ≤ 2058; third: > 2058 kcal/day

*BMI*: Body Mass Index; *CVD*: Cardiovascular Disease, including acute myocardial infarction, stroke and congestive heart failure; *GDF15*: Growth differentiation factor 15; *LDL-c*: Low-density Lipoprotein Cholesterol; *MET*: metabolic equivalent of task; *SPB*: Systolic Blood Pressure; *SPPB*: Short Physical Performance Battery

At baseline, 26.7%, 25.9%, 16.4%, 19.6% and 15.2% of study participants had reduced lower-extremity performance, impaired agility, impaired mobility, weakness and frailty, respectively (Table 2). The incidence rates of these conditions during the 2.2 year-follow-up were 22.6%, 11.7%, 9.0%, 14.0% and 10.0% respectively (Table 3). Around half of participants had at least one of the five conditions at baseline and almost 40% developed at least one of them after follow-up. There was a 27% overlap in prevalent conditions and an 11.7% overlap in incident conditions, with only 2% and 0.1%, respectively, exhibiting all five conditions (eTable 1).

**Table 2:** Odds ratios (95% confidence interval) for the cross-sectional association of GDF15 with reduced lower-extremity performance, impaired agility and mobility, weakness and frailty, at baseline

				**GDF15 (pg/mL)**		
	**Q1** (≤ 877)	**Q2** (> 877 to ≤ 1158)	**Q3** (> 1158 to ≤ 1619)	**Q4** (> 1619)	**P-trend**	**per 25% increase**
**Reduced lower-extremity performance (SPPB ≤ 9)**						
No. cases/N (%)	136/619 (21.97%)	142/620 (22.90%)	158/617 (25.61%)	225/619 (36.35%)		661/2475 (26.70%)
Model 1	Ref	0.92 (0.70; 1.21)	0.96 (0.73; 1.26)	1.49 (1.13; 1.95)**	0.003	1.09 (1.05; 1.14)***
Model 2	Ref	0.93 (0.70; 1.24)	0.91 (0.69; 1.21)	1.41 (1.06; 1.87)*	0.018	1.08 (1.03; 1.13)***
Model 3	Ref	0.93 (0.70; 1.25)	0.89 (0.66; 1.19)	1.28 (0.93; 1.75)	0.202	1.06 (1.01; 1.12)*
**Impaired agility**						
No. cases/N (%)	134/618 (21.68%)	118/614 (19.21%)	166/610 (27.21%)	216/608 (35.52%)		634/2450 (25.88%)
Model 1	Ref	0.84 (0.62; 1.12)	1.35 (1.02; 1.79)*	2.14 (1.61; 2.85)***	< 0.001	1.18 (1.13; 1.24)***
Model 2	Ref	0.83 (0.62; 1.13)	1.27 (0.95; 1.70)	2.05 (1.52; 2.76)***	< 0.001	1.17 (1.12; 1.23)***
Model 3	Ref	0.83 (0.61; 1.14)	1.27 (0.93; 1.74)	1.96 (1.39; 2.76)***	< 0.001	1.18 (1.12; 1.26)***
**Impaired mobility**						
No. cases/N (%)	81/619 (13.09%)	72/613 (11.75%)	104/609 (17.08%)	145/605 (23.97%)		402/2446 (16.43%)
Model 1	Ref	0.89 (0.62; 1.26)	1.41 (1.02; 1.97)*	2.34 (1.69; 3.25)***	< 0.001	1.20 (1.14; 1.26)***
Model 2	Ref	0.91 (0.63; 1.30)	1.35 (0.96; 1.91)	2.13 (1.51; 3.00)***	< 0.001	1.17 (1.11; 1.24)***
Model 3	Ref	0.93 (0.65; 1.35)	1.38 (0.97; 1.96)	2.11 (1.43; 3.10)***	< 0.001	1.19 (1.11; 1.27)***
**Weakness**						
No. cases/N (%)	77/618 (12.46%)	100/619 (16.16%)	119/617 (19.29%)	189/617 (30.63%)		485/2471 (19.63%)
Model 1	Ref	1.16 (0.84; 1.61)	1.26 (0.91; 1.74)	2.10 (1.54; 2.87)***	< 0.001	1.14 (1.09; 1.20)***
Model 2	Ref	1.17 (0.84; 1.63)	1.22 (0.87; 1.69)	1.90 (1.38; 2.62)***	< 0.001	1.12 (1.07; 1.17)***
Model 3	Ref	1.21 (0.87; 1.69)	1.27 (0.91; 1.78)	2.10 (1.47; 2.98)***	< 0.001	1.15 (1.09; 1.22)***
**Frailty**						
No. cases/N (%)	56/621(9.02%)	58/619 (9.37%)	90/620 (14.52%)	172/619 (27.79%)		376/2479 (15.17%)
Model 1	Ref	1.02 (0.69; 1.51)	1.65 (1.14; 2.38)**	3.90 (2.75; 5.54)***	< 0.001	1.31 (1.24; 1.38)***
Model 2	Ref	1.06 (0.70; 1.60)	1.55 (1.05; 2.29)*	3.62 (2.48; 5.27)***	< 0.001	1.28 (1.21; 1.35)***
Model 3	Ref	1.04 (0.67; 1.60)	1.45 (0.96; 2.18)	2.79 (1.81; 4.29)***	< 0.001	1.24 (1.16; 1.33)***

^*^p < 0.05; **p < 0.01; ***p < 0.001

GDF15: Growth differentiation factor 15; Q: Quartile; SPPB: Short Physical Performance Battery

Model 1: Logistic regression model adjusted for: age, sex, and education

Model 2: Further adjusted for smoking status, alcohol consumption, physical activity, time watching TV, energy intake, sleep time and diet quality (MEDAS score)

Model 3: Further adjusted for body mass index, systolic blood pressure, serum glucose, serum creatinine, serum LDL-cholesterol, cardiovascular disease, and diabetes

**Table 3:** Odds ratios (95% confidence interval) for the prospective association of GDF15 with reduced lower-extremity performance, impaired agility and mobility, weakness and frailty over 2.2 years of follow-up

	**GDF15 (pg/mL)**					
	**Q1** (≤ 877)	**Q2** (> 877 to ≤ 1158)	**Q3** (> 1158 to ≤ 1619)	**Q4** (> 1619)	**p-trend**	**per 25% increase**
**Reduced lower-extremity performance (SPPB ≤ 9)**						
No. cases/N (%)	44/316 (13.92%)	59/300 (19.67%)	73/281 (25.98%)	77/223 (34.53%)		253/1120 (22.59%)
Model 1	Ref	1.47 (0.94; 2.28)	1.74 (1.12; 2.69)*	2.66 (1.69; 4.18)***	< 0.001	1.20 (1.11; 1.30)***
Model 2	Ref	1.39 (0.89; 2.18)	1.61 (1.03; 2.53)*	2.51 (1.58; 4.00)***	< 0.001	1.19 (1.10; 1.29)***
Model 3	Ref	1.40 (0.89; 2.21)	1.62 (1.03; 2.56)*	2.37 (1.43; 3.94)***	0.001	1.18 (1.08; 1.30)***
**Impaired agility**						
No. cases/N (%)	27/359 (7.52%)	38/355 (10.70%)	43/319 (13.48%)	42/246 (17.07%)		150/1279 (11.73%)
Model 1	Ref	1.43 (0.84; 2.41)	1.73 (1.02; 2.93)*	2.39 (1.39; 4.11)**	0.001	1.17 (1.07; 1.29)***
Model 2	Ref	1.41 (0.83; 2.40)	1.70 (0.99; 2.89)	2.47 (1.42; 4.31)**	0.001	1.18 (1.07; 1.30)***
Model 3	Ref	1.39 (0.81; 2.40)	1.74 (1.00; 3.02)*	2.54 (1.37; 4.71)**	0.003	1.21 (1.08; 1.35)***
**Impaired mobility**						
No. cases/N (%)	28/401(6.98)	19/381 (4.99%)	41/357 (11.48%)	41/291 (14.09%)		129/1430 (9.02%)
Model 1	Ref	0.69 (0.38; 1.28)	1.64 (0.96; 2.78)	2.24 (1.30; 3.86)**	< 0.001	1.21 (1.10; 1.33)***
Model 2	Ref	0.66 (0.36; 1.23)	1.54 (0.90; 2.65)	2.24 (1.28; 3.91)**	< 0.001	1.21 (1.10; 1.33)***
Model 3	Ref	0.69 (0.37; 1.30)	1.62 (0.93; 2.82)	2.20 (1.19; 4.08)*	0.002	1.21 (1.08; 1.35)***
**Weakness**						
No. cases/N (%)	43/352 (12.22%)	43/325 (13.23%)	44/302 (14.57%)	41/238 (17.22%)		171/1217 (14.05%)
Model 1	Ref	1.04 (0.65; 1.64)	1.04 (0.65; 1.66)	1.16 (0.71; 1.90)	0.582	1.05 (0.97; 1.14)
Model 2	Ref	1.02 (0.64; 1.62)	0.98 (0.61; 1.58)	1.17 (0.71; 1.94)	0.615	1.06 (0.97; 1.15)
Model 3	Ref	1.08 (0.67; 1.73)	1.08 (0.66; 1.75)	1.53 (0.87; 2.67)	0.199	1.13 (1.02; 1.25)*
**Frailty**						
No. cases/N (%)	30/420 (7.14%)	31/399 (7.77%)	38/372 (10.21%)	49/289 (16.96%)		148/1480 (10.00%)
Model 1	Ref	1.13 (0.66; 1.92)	1.44 (0.85; 2.43)	2.82 (1.68; 4.74)***	< 0.001	1.23 (1.13; 1.35)***
Model 2	Ref	1.08 (0.62; 1.86)	1.37 (0.80; 2.35)	2.73 (1.59; 4.68)***	< 0.001	1.22 (1.11; 1.33)***
Model 3	Ref	1.07 (0.61; 1.88)	1.24 (0.70; 2.18)	2.29 (1.26; 4.18)**	0.008	1.18 (1.06; 1.31)**

^*^p < 0.05; **p < 0.01; ***p < 0.001

GDF15: Growth differentiation factor 15; Q:Quartile; SPPB: Short Physical Performance Battery

Model 1: Logistic regression model adjusted for: age, sex, and education

Model 2: Further adjusted for smoking status, alcohol consumption, physical activity, time watching TV, energy intake, sleep time and diet quality (MEDAS score)

Model 3: Further adjusted for body mass index, systolic blood pressure, serum glucose, serum creatinine, serum LDL-cholesterol, cardiovascular disease, and diabetes

**Fig. 1:**
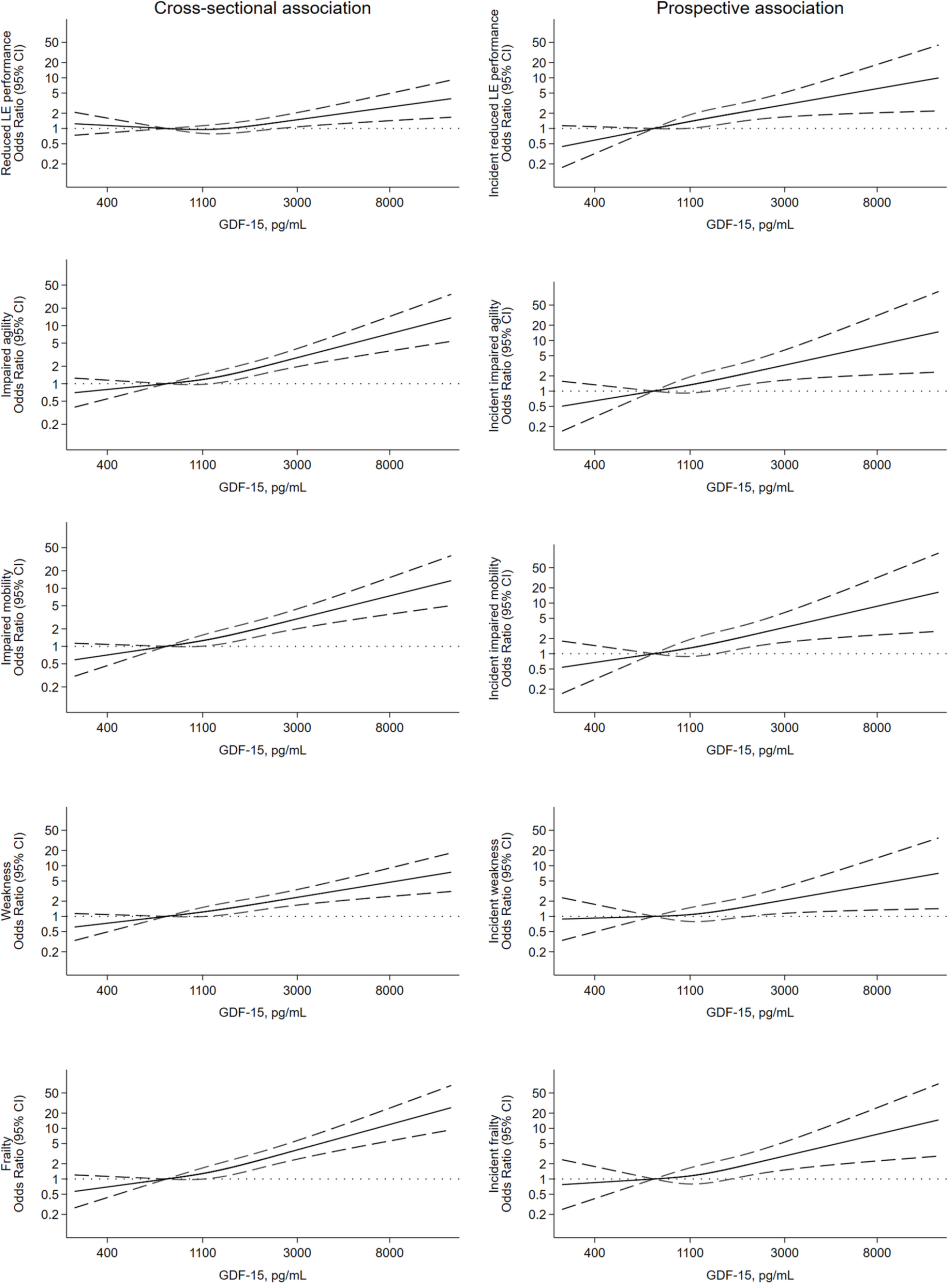
Cross-sectional and prospective association of GDF15 with impaired physical function. CI: confidence interval; GDF15: Growth differentiation factor 15; LE: lower-extremity. Restricted cubic splines (knots at the 10th, 50th and 90th percentile of GDF15 concentration; reference at the median GDF15 concentration for the lowest quartile) from logistic regression models adjusted for: age, sex, education, smoking status, alcohol consumption, physical activity, time watching TV, energy intake, sleep time, diet quality (MEDAS score), body mass index, systolic blood pressure, serum glucose, serum creatinine, serum LDL-cholesterol, cardiovascular disease, and diabetes This figure was created with Stata®, version 15 (StataCorp)

In multivariable analyses, higher GDF15 serum levels showed a dose–response association with a higher frequency of reduced lower-extremity performance, impaired agility and mobility, weakness and frailty at baseline and over a 2.2-year follow-up (Tables 2 and 3, Fig. 1).

In the cross-sectional analysis, for the fully adjusted model, a 25% increment in baseline GDF15 levels showed OR (95% CI) of 1.06 (1.01; 1.12) for reduced lower-extremity performance (SPPB ≤ 9), 1.18 (1.12; 1.26) for impaired agility, 1.19 (1.11; 1.27) for impaired mobility, 1.15 (1.09; 1.22) for weakness and 1.24 (1.16; 1.33) for frailty (Table 2). After a mean follow-up of 2.2 years, a 25% higher baseline GDF15 concentration was also associated with an increased risk of reduced lower-extremity performance, as well as impaired agility and mobility, weakness and frailty, with OR (95% CI) of 1.18 (1.08; 1.30), 1.21 (1.08; 1.35), 1.21 (1.08; 1.35), 1.13 (1.02; 1.25), and 1.18 (1.06; 1.31), respectively (Table 3).

In terms of the SPPB components, the cross-sectional OR (95% CI) for reduced performance in the balance, gait-speed and sit-to-stand tests were 1.12 (1.05; 1.20), 1.03 (0.98; 1.08), and 1.06 (1.00; 1.12), respectively. In the prospective analysis, the OR (95% CI) were 1.07 (0.96; 1.20), 1.15 (1.06; 1.26), and 1.16 (1.02; 1.31), respectively (eTable 2), corroborating the consistency of the associations.

As regards sensitivity analyses, further adjustment for depression, cognitive deterioration, appetite or IL-6 levels at baseline did not materially modify the results (eTables 3 and 4). Results were also similar when participants with CVD or diabetes were excluded from the analyses, although the cross-sectional association with the SPPB score and the prospective one with agility impairment and weakness fell short of statistical significance (eTables 5 and 6). Consistent findings were also observed when modeling lower-extremity performance, weakness, and frailty as continuous variables (eTables 7 and 8).

## Discussion

In older adults, higher serum GDF15 concentrations were associated with reduced lower-extremity performance, impaired agility and mobility, as well as an increased risk of weakness and frailty, independently of sociodemographic, lifestyle and clinical characteristics, both at baseline and after 2.2 years of follow-up. After excluding participants with CVD or diabetes, most associations remained. These findings provide evidence that serum GDF15 is not only associated with poor physical function cross-sectionally but may also serve as a longitudinal indicator of emerging functional decline. This supports our initial hypothesis that GDF15 captures early biological changes relevant to aging, rather than being merely a correlate of disease burden or comorbidity.

Some studies have already shown consistent associations between GDF15 and muscular function [26–28], suggesting that GDF15 pathways play a role in skeletal muscle health. There are also studies examining the associations of GDF15 with physical performance [25, 29, 55]. In line with our results, a study examining the association between GDF15 and grip strength and lower-limb physical performance [26] using the SPPB score and its components demonstrated an inverse cross-sectional association of GDF15 with grip strength, the global SPPB score and the gait speed score, but not with the sit-to-stand test. However, no prospective associations were observed, in contrast with our data. Furthermore, our study is the first to combine the assessment of the SPPB score and its components with the evaluation of mobility and agility, in order to develop a more robust analysis of impaired leg function—factors that the previous study acknowledged in its conclusions, emphasizing the need for further investigation. We have also examined the relationship between GDF15 and the risk of frailty by using a DAI, a tool also often utilized in comprehensive geriatric assessments. Previous research has found cross-sectional associations of GDF15 with frailty in older adults admitted acutely [56], and with cognitive frailty [57], and a longitudinal association with the onset of frailty in older adults with cardiometabolic diseases [58]. However, to our knowledge, there are no studies evaluating the prospective association of GDF15 and frailty in community-dwelling older adults. Therefore, our study adds significant robustness and depth to the existing evidence on the association between GDF15 and frailty and, ultimately, physical function impairment in older adults, regardless of diabetes or CVD. Although age, comorbidities, and baseline function are strong predictors of future physical decline, GDF15 remained independently associated with multiple domains of impairment after adjusting for these variables. This suggests that GDF15 captures additional biological vulnerability to function decline not explained by clinical or behavioral variables alone.

The biological mechanisms by which upregulation of GDF15 could negatively impact skeletal muscle health are still unclear. A possible mechanism linking increased GDF15 levels with age-related impaired neuromuscular function might be the elevation of mammalian target of rapamycin complex 1 (mTORC1) in Akt-independent activation in ageing, which regulates GDF15 gene expression in humans, while upregulating autophagic marker LC3 and inducing increases in protein ubiquitination and oxidation [27]. This process leads to degenerative changes, such as muscle atrophy, loss of type II fibers, mitochondrial dysfunction, and reductions in maximal isometric muscle force production and exercise capacity [27].

Another possible contributor to the decline in physical function in older adults is anorexia, which is common and may be related to several factors, such as reduced smell and taste, changes in gastrointestinal function, hormonal alterations, polypharmacy, and limitations in daily activities [59]. Anorexia may also be due to a negative balance between the reduction in energy intake that occurs from the third to the ninth decade of life and the decrease of energy expenditure, also frequent as age increases [60]. Some studies have reported that adults with anorexia are at higher risk of disability [61], whereas other showed an association between anorexia and sarcopenia measured through physical performance scores [61]. In this regard, the recent discovery of the signaling axis GDF15–GFRAL (glial cell-derived neurotrophic factor family receptor alpha-like) receptor provides a potential mechanism for the downregulation of food intake in older adults [28, 30]. Studies in mice demonstrated that elevations in GDF15 reduced food intake and body mass through binding to GFRAL and recruitment of the receptor tyrosine kinase RET in the hindbrain, independently of other appetite-regulating hormones [31]. While GDF15 may not be a direct cause of impairment, its role in signaling mitochondrial stress and activating the GFRAL–RET pathway, which suppresses appetite and promotes muscle catabolism, combined with its implication in the mTORC1-dependent pathways, likely reflects upstream mechanisms contributing to age-related functional loss and provide biological plausibility of GDF15 as a marker of functional decline. Therefore, there is a growing interest in using GDF15 as a target to treat not only many metabolic diseases, such as obesity, diabetes, CVD, and cancer, but also to prevent cachexia and other age-related disorders. However, there are some inconsistences between animal and human studies. For example, while mice models overexpressing GDF15 improved glucose tolerance [62] and insulin sensitivity [63], in humans high levels of GDF15 were linked to impaired glucose tolerance and insulin resistance [64]. From this perspective, transient peaks in GDF15 may be beneficial, while chronically elevated systemic levels are detrimental to skeletal muscle homeostasis and neuromuscular function. The association between elevated GDF15 and impaired physical function may, therefore, reflect several underlying mechanisms: the stress response pathway and signals through the GFRAL–RET receptor, the mTORC1-dependent pathways, and the mitochondrial and metabolic stress. While we did not directly assess these mechanisms, the observed associations across multiple functional domains support the hypothesis that GDF15 is a sensitive marker of multisystem dysregulation relevant to age-related functional decline. The longitudinal design of our study supports the hypothesis that elevated GDF15 may precede physical function loss, aligning with prior findings that associate GDF15 with early biological stress responses and vulnerability in aging.

A recently published phase 2 clinical study demonstrated that the inhibition of GDF15 in patients with cancer cachexia and an elevated serum GDF15 levels resulted in increased weight gain and overall activity level, with reduced cachexia symptoms [65], corroborating its therapeutic potential but reinforcing the need to clarify the mechanisms of GDF15 associated with aging, disability, and frailty, in order to expand the therapeutic utility.

This study has several strengths. First, to our knowledge, it is the first evaluation to date of the specific prospective association between GDF15 concentration and the risk of impaired physical function based simultaneously on five validated scores in community-dwelling older adults. Although serum GDF15 was measured only at baseline, physical function outcomes were assessed both cross-sectionally and after a median follow-up of 2.2 years, allowing us to evaluate the potential predictive role of GDF15 in functional decline. Second, we performed interaction analysis to assess the robustness of our results across sex and age and minimized reverse causation by excluding individuals with diabetes and CVD. These conditions are known to elevate GDF15 levels and contribute to functional impairment. Their exclusion allowed us to assess whether GDF15 predicts functional decline in participants without overt chronic disease, suggesting its potential as a marker of early biological aging rather than disease burden. Finally, we applied different methods to model the dose–response relationship between GDF15 and functional variables. Our combined use of categorical and continuous outcome modeling was intentional and aligned with both clinical practice and biomarker epidemiology literature. Logistic regression allowed us to estimate the odds of crossing clinically meaningful thresholds of functional impairment, facilitating real-world applicability. Complementary linear regression analyses using continuous outcomes yielded similar associations, confirming that our findings are not dependent on categorization alone. However, our study also has some limitations. Indeed, there may have been some measurement error in GDF15, which might have attenuated the true associations. Nonetheless the error is likely to be small, because the measurement kit has been validated, and the inter-assay coefficient of variation is also fairly small. Also, as in any observational study, we cannot entirely rule out some residual confounding, despite adjusting for many potential confounders, including important determinants of muscular function, like diet quality, appetite, and physical activity, as well as correlates of disease burden such as BMI, SBP, serum glucose, serum LDL-c, serum creatinine, CVD and diabetes, and biomarkers of inflammation, such as serum IL-6 levels. Moreover, the similarity of associations across different physical function outcomes could partly reflect the interrelated nature of mobility, strength, and frailty domains, although there was not much overlap in impairments, especially at follow-up.

In conclusion, our results provide evidence that at higher levels of GDF15 there is a higher risk of impaired physical function in older adults. From a clinical standpoint, GDF15 could serve as a convenient biomarker to identify older adults at risk of physical function decline—even in the absence of overt disease or frailty. This could support early interventions, such as physical therapy, nutritional support, or closer functional monitoring. Future research should elucidate the biological mechanisms underlying the observed associations to explore all the therapeutic potential of modifying GDF15, and evaluate whether incorporating GDF15 into prognostic models can improve risk prediction and care planning in aging populations.

## Supplementary Information

Below is the link to the electronic supplementary material.

Supplementary file1 (PDF 671 KB)

## Data Availability

The datasets used and/or analyzed during the current study are available from the corresponding author on reasonable request.
