# Supplementary material for: Growth differentiation factor 15 predicts physical function impairment in Spanish older adults: a real-world prospective study
Source: GeroScience. 2025 Jul 2;48(2):1799–814. doi: 10.1007/s11357-025-01779-3 (PMC12972462; doi:10.1007/s11357-025-01779-3)
Supplement: Supplementary file 1 — Supplementary file1 (PDF 671 KB) [file 11357_2025_1779_MOESM1_ESM.pdf]

eTable 1. Distribution of participants according to the number of coexistent conditions at baseline and after 2.2-years of follow-up.

Number of coexistent conditions	Prevalent conditions at baseline (n=2481) N (%)	Incident conditions over the 2.2-year follow-up (n=1649) N (%)
0	1218 (49.1%)	1031 (62.5%)
1	583 (23.9%)	442 (26.8%)
2	277 (11.2%)	126 (7.6%)
3	211 (8.5%)	44 (2.7%)
4	132 (5.3%)	5 (0.3%)
5	50 (2.0%)	1 (0.1%)

eTable 2. Odds ratios (95% confidence interval) for the cross-sectional and prospective associations of GDF15 with the SPPB components.

	Q1 (≤ 877)	Q2 (> 877 to ≤ 1158)	Q3 (> 1158 to ≤ 1619)	Q4 (> 1619)	P-trend	per 25% increase
**Cross-sectional Analysis**						
	**GDF15 (pg/mL)**					
**Balance score**						
No. cases/N (%)	49/619 (7.91%)	57/620 (9.19%)	81/617 (13.13%)	128/619 (20.68%)		315/2475 (12.73%)
Model 1	Ref.	1.02 (0.67; 1.53)	1.35 (0.91; 2.00)	2.12 (1.45; 3.10)***	<0.001	1.17 (1.10; 1.23)***
Model 2	Ref.	1.00 (0.66; 1.52)	1.22 (0.82; 1.83)	1.84 (1.24; 2.73)**	<0.001	1.14 (1.07; 1.20)***
Model 3	Ref.	1.01 (0.66; 1.55)	1.20 (0.79; 1.82)	1.65 (1.07; 2.55)*	0.013	1.12 (1.05; 1.20)***
**Gait Speed score**						
No. cases/N (%)	177/619 (28.59%)	170/620 (27.42%)	184/617 (29.82%)	216/619 (34.89%)		747/2475 (30.18%)
Model 1	Ref.	0.88 (0.68; 1.13)	0.93 (0.72; 1.20)	1.12 (0.87; 1.45)	0.305	1.04 (0.99; 1.08)
Model 2	Ref.	0.90 (0.69; 1.16)	0.93 (0.72; 1.20)	1.10 (0.84; 1.43)	0.433	1.03 (0.99; 1.08)
Model 3	Ref.	0.91 (0.70; 1.18)	0.93 (0.72; 1.21)	1.08 (0.81; 1.44)	0.627	1.03 (0.98; 1.08)
**Sit-to-Stand score**						
No. cases/N (%)	443/619 (71.57%)	474/620 (76.45%)	483/617 (78.28%)	505/619 (81.58%)		1908/2475 (77.09%)
Model 1	Ref.	1.21 (0.93; 1.57)	1.29 (0.99; 1.69)	1.47 (1.11; 1.96)**	0.007	1.08 (1.03; 1.14)***
Model 2	Ref.	1.17 (0.90; 1.52)	1.23 (0.94; 1.62)	1.39 (1.04; 1.86)*	0.024	1.07 (1.02; 1.13)**
Model 3	Ref.	1.13 (0.86; 1.47)	1.16 (0.88; 1.54)	1.21 (0.87; 1.67)	0.234	1.06 (1.00; 1.12)
**Prospective Analysis**						
	**GDF15 (pg/mL)**					
	**Q1** (≤ 877)	**Q2** (> 877 to ≤ 1158)	**Q3** (> 1158 to ≤ 1619)	**Q4** (> 1619)	**P-trend**	**per 25% increase**
**Balance score**						
No. cases/N (%)	25/368 (6.79%)	28/353 (7.93%)	29/324 (8.95%)	42/274 (15.33%)		124/1319 (9.40%)
Model 1	Ref.	1.12 (0.63; 1.98)	1.07 (0.60; 1.91)	1.90 (1.08; 3.32)*	0.032	1.11 (1.01; 1.22)*
Model 2	Ref.	1.10 (0.61; 1.97)	1.02 (0.57; 1.85)	1.76 (0.99; 3.14)	0.071	1.09 (0.99; 1.20)
Model 3	Ref.	1.10 (0.61; 1.99)	1.03 (0.57; 1.88)	1.69 (0.89; 3.19)	0.152	1.07 (0.96; 1.20)
**Gait Speed score**						
No. cases/N (%)	66/287 (22.99%)	71/274 (25.91%)	91/254 (35.83%)	97/220 (44.09%)		325/1035 (31.40%)
Model 1	Ref.	1.06 (0.71; 1.59)	1.39 (0.93; 2.09)	2.02 (1.33; 3.07)***	<0.001	1.18 (1.10; 1.27)***
Model 2	Ref.	1.01 (0.67; 1.52)	1.30 (0.86; 1.97)	1.85 (1.21; 2.83)**	0.002	1.16 (1.08; 1.25)***
Model 3	Ref.	1.00 (0.66; 1.51)	1.24 (0.81; 1.90)	1.70 (1.07; 2.72)*	0.019	1.15 (1.06; 1.26)**
**Sit-to-Stand score**						
No. cases/N (%)	44/121 (36.36%)	41/98 (41.84%)	56/97 (57.73%)	31/69 (44.93%)		172/385 (44.68%)
Model 1	Ref.	1.25 (0.72; 2.17)	2.29 (1.31; 4.02)**	1.36 (0.72; 2.56)	0.061	1.12 (1.01; 1.25)*
Model 2	Ref.	1.22 (0.68; 2.18)	2.13 (1.19; 3.83)*	1.33 (0.68; 2.61)	0.094	1.11 (0.99; 1.24)
Model 3	Ref.	1.25 (0.68; 2.27)	2.31 (1.26; 4.22)**	1.49 (0.70; 3.15)	0.046	1.16 (1.02; 1.31)*

***p<0.05; **p<0.01; ***p<0.001**

GDF15: Growth differentiation factor 15; **Q**: Quartile; **SPPB:** Short Physical Performance Battery

Model 1: Logistic regression model adjusted for: age, sex, and education.

Model 2: Further adjusted for smoking status, alcohol consumption, physical activity, time watching TV, energy intake, sleep time and diet quality (MEDAS score).

Model 3: Further adjusted for body mass index, systolic blood pressure, serum glucose, serum creatinine, serum LDL-cholesterol, cardiovascular disease, and diabetes.

eTable 3. Sensitivity analyses. Odds ratios (95% confidence interval) for the cross-sectional association of GDF15 with the risk of impaired physical function at baseline, with further adjustment for confounders with respect to the main analyses[ǂ].

	GDF-15 (pg/mL)					
	Q1 (≤ 877)	Q2 (> 877 to ≤ 1158)	Q3 (> 1158 to ≤ 1619)	Q4 (> 1619)	P-trend	per 25% increase
**Reduced lower-extremity performance (SPPB ≤9)**						
No. Cases/N (%)	136/619 (21.97%)	142/620 (22.90%)	158/617 (25.61%)	225/619 (36.35%)		661/2475 (26.71%)
Model 3	Ref.	0.93 (0.70; 1.25)	0.89 (0.66; 1.19)	1.28 (0.93; 1.75)	0.202	1.06 (1.01; 1.12)*
+ depression	Ref.	0.93 (0.70; 1.25)	0.88 (0.66; 1.18)	1.27 (0.92; 1.75)	0.215	1.06 (1.01; 1.12)*
+ cognitive deterioration	Ref.	0.93 (0.70; 1.24)	0.88 (0.66; 1.18)	1.24 (0.90; 1.71)	0.267	1.06 (1.00; 1.12)*
+ appetite	Ref.	0.93 (0.70; 1.25)	0.88 (0.66; 1.19)	1.24 (0.90; 1.71)	0.274	1.06 (1.00; 1.12)*
+ IL-6	Ref.	0.92 (0.69; 1.23)	0.87 (0.65; 1.17)	1.20 (0.87; 1.66)	0.370	1.05 (1.00; 1.11)
**Impaired agility**						
No. Cases/N (%)	134/618 (21.68%)	118/614 (19.22%)	166/610 (27.21%)	216/608 (35.53%)		634/2450 (25.88%)
Model 3	Ref.	0.83 (0.61; 1.14)	1.27 (0.93; 1.74)	1.96 (1.39; 2.76)***	<0.001	1.18 (1.12; 1.26)***
+ depression	Ref.	0.83 (0.61; 1.15)	1.23 (0.90; 1.69)	1.93 (1.37; 2.72)***	<0.001	1.18 (1.12; 1.26)***
+ cognitive deterioration	Ref.	0.83 (0.60; 1.14)	1.24 (0.91; 1.70)	1.92 (1.36; 2.72)***	<0.001	1.18 (1.11; 1.25)***
+ appetite	Ref.	0.83 (0.60; 1.15)	1.24 (0.91; 1.70)	1.92 (1.36; 2.72)***	<0.001	1.18 (1.11; 1.25)***
+ IL-6	Ref.	0.83 (0.60; 1.14)	1.22 (0.89; 1.68)	1.86 (1.31; 2.64)***	<0.001	1.17 (1.11; 1.25)***
**Impaired mobility**						
No. Cases/N (%)	81/619 (13.08%)	72/613 (15.32%)	104/609 (23.22%)	145/605 (40.50%)		402/2446 (16.34%)
Model 3	Ref.	0.93 (0.65; 1.35)	1.38 (0.97; 1.96)	2.11 (1.43; 3.10)***	<0.001	1.19 (1.11; 1.27)***
+ depression	Ref.	0.94 (0.65; 1.36)	1.34 (0.94; 1.91)	2.08 (1.41; 3.06)***	<0.001	1.19 (1.11; 1.27)***
+ cognitive deterioration	Ref.	0.93 (0.64; 1.34)	1.33 (0.93; 1.90)	2.02 (1.37; 2.98)***	<0.001	1.18 (1.11; 1.26)***
+ appetite	Ref.	0.94 (0.65; 1.36)	1.35 (0.94; 1.92)	2.04 (1.38; 3.01)***	<0.001	1.18 (1.11; 1.26)***
+ IL-6	Ref.	0.89 (0.61; 1.30)	1.29 (0.90; 1.85)	1.88 (1.27; 2.79)**	<0.001	1.16 (1.09; 1.24)***
**Weakness**						
No. Cases/N (%)	77/618 (12.46%)	100/619 (8.40%)	119/617 (11.67%)	189/617 (31.66%)		485/2471 (15.18%)
Model 3	Ref.	1.21 (0.87; 1.69)	1.27 (0.91; 1.78)	2.10 (1.47; 2.98)***	<0.001	1.15 (1.09; 1.22)***
+ depression	Ref.	1.21 (0.87; 1.69)	1.25 (0.89; 1.75)	2.08 (1.46; 2.96)***	<0.001	1.15 (1.08; 1.22)***
+ cognitive deterioration	Ref.	1.20 (0.85; 1.67)	1.25 (0.89; 1.75)	2.04 (1.43; 2.91)***	<0.001	1.15 (1.08; 1.21)***
+ appetite	Ref.	1.19 (0.85; 1.67)	1.24 (0.89; 1.74)	2.02 (1.42; 2.89)***	<0.001	1.14 (1.08; 1.21)***
+ IL-6	Ref.	1.17 (0.84; 1.64)	1.21 (0.86; 1.70)	1.93 (1.35; 2.76)***	<0.001	1.13 (1.07; 1.20)***
**Frailty**						
No. Cases/N (%)	56/621 (9.02%)	58/619 (8.40%)	90/620 (11.67%)	172/619 (31.66%)		376/2479 (15.18%)
Model 3	Ref.	1.04 (0.67; 1.60)	1.45 (0.96; 2.18)	2.79 (1.81; 4.29)***	<0.001	1.24 (1.16; 1.33)***
+ depression	Ref.	1.07 (0.69; 1.66)	1.35 (0.89; 2.06)	2.77 (1.78; 4.31)***	<0.001	1.24 (1.16; 1.33)***
+ cognitive deterioration	Ref.	1.07 (0.68; 1.67)	1.34 (0.87; 2.05)	2.66 (1.70; 4.15)***	<0.001	1.23 (1.15; 1.32)***
+ appetite	Ref.	1.06 (0.68; 1.65)	1.32 (0.86; 2.02)	2.62 (1.67; 4.09)***	<0.001	1.23 (1.15; 1.32)***
+ IL-6	Ref.	1.04 (0.67; 1.64)	1.26 (0.82; 1.93)	2.40 (1.53; 3.77)***	<0.001	1.21 (1.13; 1.30)***

***p<0.05; **p<0.01; ***p<0.001**

GDF15: Growth differentiation factor 15; **Q**: Quartile; **SPPB:** Short Physical Performance Battery

Model 3: Logistic regression model adjusted for: age, sex, education, smoking status, alcohol consumption, physical activity, time watching TV, energy intake, sleep time, diet quality (MEDAS score), body mass index, systolic blood pressure, serum glucose, serum creatinine, serum LDL-cholesterol, cardiovascular disease, and diabetes.

[ǂ] **Further adjustment:** depression requiring treatment, cognitive deterioration (Mini-Mental State Examination<24) appetite (very poor, poor, rather good, very good), and interleukin-6 (IL-6) levels (quartiles) at baseline.

eTable 4. Sensitivity analyses. Odds ratios (95% confidence interval) for the prospective association of GDF15 with the risk of impaired physical function over 2.2 years of follow-up, with further adjustment for confounders with respect to the main analyses[ǂ].

	GDF-15 (pg/mL)					
	**Q1** (≤ 877)	**Q2** (> 879 to ≤ 1158)	**Q3** (> 1158 to ≤ 1619)	**Q4** (> 1619)	**P-trend**	**per 25% increase**
**Reduced lower-extremity performance (SPPB ≤9)**						
No. Cases/N (%)	44/316 (13.92%)	59/300 (19.67%)	73/281 (25.98%)	77/223 (34.53%)		253/1120 (22.59%)
Model 3	Ref.	1.40 (0.89; 2.21)	1.62 (1.03; 2.56)*	2.37 (1.43; 3.94)***	<0.001	1.18 (1.08; 1.30)***
+ depression	Ref.	1.40 (0.88; 2.20)	1.60 (1.01; 2.52)*	2.31 (1.39; 3.84)**	<0.001	1.18 (1.08; 1.29)***
+ cognitive deterioration	Ref.	1.40 (0.88; 2.21)	1.62 (1.02; 2.57)*	2.34 (1.40; 3.91)**	<0.001	1.18 (1.08; 1.30)***
+ appetite	Ref.	1.38 (0.87; 2.19)	1.63 (1.03; 2.59)*	2.32 (1.39; 3.87)**	<0.001	1.18 (1.08; 1.29)***
+ IL-6	Ref.	1.32 (0.83; 2.09)	1.54 (0.97; 2.45)	2.12 (1.26; 3.55)**	0.004	1.16 (1.06; 1.27)**
**Impaired agility**						
No. Cases/N (%)	27/359 (7.52%)	38/355 (10.70%)	43/319 (13.48%)	42/246 (17.07%)		150/1279 (11.73%)
Model 3	Ref.	1.39 (0.81; 2.40)	1.74 (1.00; 3.02)*	2.54 (1.37; 4.71)**	0.003	1.21 (1.08; 1.35)***
+ depression	Ref.	1.37 (0.79; 2.36)	1.71 (0.98; 2.97)	2.48 (1.34; 4.61)**	0.003	1.20 (1.08; 1.35)**
+ cognitive deterioration	Ref.	1.36 (0.79; 2.35)	1.72 (0.99; 2.99)	2.52 (1.36; 4.68)**	0.003	1.21 (1.08; 1.35)***
+ appetite	Ref.	1.36 (0.79; 2.35)	1.72 (0.99; 3.00)	2.52 (1.36; 4.69)**	0.003	1.21 (1.08; 1.35)***
+ IL-6	Ref.	1.35 (0.78; 2.34)	1.71 (0.98; 2.99)	2.49 (1.34; 4.64)**	0.003	1.21 (1.08; 1.35)**
**Impaired mobility**						
No. Cases/N (%)	28/401 (6.98%)	19/381 (4.99%)	41/357 (11.48%)	41/291 (14.09%)		129/1430 (9.02%)
Model 3	Ref.	0.69 (0.37; 1.30)	1.62 (0.93; 2.82)	2.20 (1.19; 4.08)*	0.002	1.21 (1.08; 1.35)***
+ depression	Ref.	0.68 (0.36; 1.29)	1.56 (0.89; 2.72)	2.12 (1.14; 3.95)*	0.003	1.21 (1.08; 1.35)**
+ cognitive deterioration	Ref.	0.67 (0.36; 1.27)	1.56 (0.89; 2.73)	2.07 (1.11; 3.86)*	0.004	1.20 (1.07; 1.34)**
+ appetite	Ref.	0.70 (0.37; 1.32)	1.59 (0.91; 2.80)	2.19 (1.17; 4.10)*	0.002	1.21 (1.08; 1.35)***
+ IL-6	Ref.	0.69 (0.36; 1.30)	1.56 (0.89; 2.74)	2.09 (1.12; 3.93)*	0.004	1.20 (1.07; 1.35)**
**Weakness**						
No. Cases/N (%)	43/352 (12.22%)	43/325 (13.23%)	44/302 (14.57%)	41/238 (17.23%)		171/1217 (14.05%)
Model 3	Ref.	1.08 (0.67; 1.73)	1.08 (0.66; 1.75)	1.53 (0.87; 2.67)	0.199	1.13 (1.02; 1.25)*
+ depression	Ref.	1.10 (0.68; 1.76)	1.06 (0.65; 1.73)	1.48 (0.84; 2.59)	0.251	1.12 (1.02; 1.24)*
+ cognitive deterioration	Ref.	1.10 (0.68; 1.76)	1.07 (0.66; 1.75)	1.46 (0.83; 2.56)	0.265	1.12 (1.01; 1.24)*
+ appetite	Ref.	1.09 (0.68; 1.75)	1.07 (0.66; 1.74)	1.43 (0.81; 2.50)	0.293	1.12 (1.01; 1.24)*
+ IL-6	Ref.	1.07 (0.67; 1.72)	1.05 (0.65; 1.72)	1.39 (0.79; 2.44)	0.336	1.11 (1.01; 1.23)*
**Frailty**						
No. Cases/N (%)	30/420 (7.14%)	31/399 (7.77%)	38/372 (10.22%)	49/289 (16.96%)		148/1480 (10.00%)
Model 3	Ref.	1.07 (0.61; 1.88)	1.24 (0.70; 2.18)	2.29 (1.26; 4.18)**	0.008	1.18 (1.06; 1.31)**
+ depression	Ref.	1.10 (0.62; 1.95)	1.22 (0.69; 2.17)	2.32 (1.26; 4.28)**	0.009	1.19 (1.07; 1.32)**
+ cognitive deterioration	Ref.	1.10 (0.62; 1.94)	1.22 (0.69; 2.17)	2.32 (1.26; 4.28)**	0.009	1.19 (1.07; 1.32)**
+ appetite	Ref.	1.05 (0.59; 1.87)	1.17 (0.66; 2.09)	2.13 (1.15; 3.95)*	0.020	1.17 (1.05; 1.31)**
+ IL-6	Ref.	1.05 (0.59; 1.86)	1.17 (0.65; 2.09)	2.12 (1.14; 3.95)*	0.020	1.17 (1.05; 1.31)**

***p<0.05; **p<0.01; ***p<0.001**

GDF-15: Growth differentiation factor 15; **Q**: Quartile; **SPPB:** Short Physical Performance Battery

Model 3: Logistic regression model adjusted for: age, sex, education, smoking status, alcohol consumption, physical activity, time watching TV, energy intake, sleep time, diet quality (MEDAS score), body mass index, systolic blood pressure, serum glucose, serum creatinine, serum LDL-cholesterol, cardiovascular disease, and diabetes.

[ǂ] **Further adjustments:** depression requiring treatment, cognitive deterioration (Mini-Mental State Examination <24) appetite (very poor, poor, rather good, very good), and interleukin-6 (IL-6) levels (quartiles) at baseline.

eTable 5. Odds ratios (95% confidence interval) for the cross-sectional association of GDF15 with risk of reduced lower-extremity performance, impaired agility and mobility, weakness and frailty, at baseline, excluding participants with cardiovascular disease and/or diabetes.

	GDF15 (pg/mL)					
	**Q1** (≤ 846)	**Q2** (>846 to ≤ 1086)	**Q3** (>1086 to ≤1422)	**Q4** (> 1422)	**p-trend**	**per 25% increase**
**Reduced lower-extremity performance (SPPB ≤9)**						
No. cases/N (%)	119/564 (21.10%)	119/538 (22.13%)	115/493 (23.32%)	109/328 (33.23%)		462/1923 (24.02%)
Model 1	Ref.	0.92 (0.68; 1.23)	0.87 (0.64; 1.18)	1.24 (0.89; 1.74)	0.388	1.06 (0.99; 1.12)
Model 2	Ref.	0.93 (0.69; 1.26)	0.82 (0.59; 1.12)	1.19 (0.84; 1.68)	0.670	1.04 (0.98; 1.11)
Model 3	Ref.	0.94 (0.69; 1.28)	0.83 (0.60; 1.14)	1.23 (0.85; 1.78)	0.612	1.05 (0.98; 1.13)
**Impaired agility**						
No. cases/N (%)	111/562 (19.75%)	88/535 (16.45%)	132/490 (26.94%)	111/326 (34.05%)		442/1913 (23.11%)
Model 1	Ref.	0.78 (0.56; 1.08)	1.54 (1.13; 2.11)**	2.24 (1.57; 3.19)***	<0.001	1.21 (1.13; 1.29)***
Model 2	Ref.	0.79 (0.56; 1.10)	1.47 (1.06; 2.04)*	2.22 (1.53; 3.22)***	<0.001	1.20 (1.12; 1.28)***
Model 3	Ref.	0.82 (0.57; 1.16)	1.62 (1.15; 2.28)**	2.74 (1.82; 4.11)***	<0.001	1.27 (1.18; 1,38)***
**Impaired mobility**						
No. cases/N (%)	72/563 (12.79%)	53/534 (9.93%)	80/490 (16.33%)	73/325 (22.46%)		278/1912 (14.54%)
Model 1	Ref.	0.75 (0.51; 1.11)	1.38 (0.95; 1.99)	2.12 (1.41; 3.16)***	<0.001	1.18 (1.09; 1.27)***
Model 2	Ref.	0.76 (0.51; 1.14)	1.29 (0.88; 1.89)	2.00 (1.31; 3.06)**	<0.001	1.15 (1.07; 1.25)***
Model 3	Ref.	0.79 (0.52; 1.20)	1.41 (0.95; 2.09)	2.45 (1.55; 3.87)***	<0.001	1.21 (1.11; 1.32)***
**Weakness**						
No. cases/N (%)	67/563 (11.90%)	82/538 (15.25%)	92/493 (18.66%)	104/327 (31.80%)		345/1921(17.96%)
Model 1	Ref.	1.19 (0.84; 1.69)	1.37 (0.96; 1.96)	2.51 (1.74; 3.63)***	<0.001	1.19 (1.11; 1.27)***
Model 2	Ref.	1.19 (0.83; 1.71)	1.30 (0.90; 1.86)	2.24 (1.53; 3.28)***	<0.001	1.16 (1.08; 1.24)***
Model 3	Ref.	1.24 (0.86; 1.77)	1.37 (0.95; 1.98)	2.57 (1.72; 3.83)***	<0.001	1.19 (1.11; 1.28)***
**Frailty**						
No. cases/N (%)	42/565 (7.43%)	39/538 (7.25%)	65/494 (13.16%)	70/329 (21.28%)		216/1926 (11.22%)
Model 1	Ref.	0.97 (0.61; 1.55)	1.93 (1.26; 2.95)**	3.55 (2.27; 5.55)***	<0.001	1.29 (1.19; 1.40)***
Model 2	Ref.	1.01 (0.62; 1.63)	1.79 (1.14; 2.81)*	3.31 (2.04; 5.37)***	<0.001	1.24 (1.14; 1.35)***
Model 3	Ref.	1.07 (0.65; 1.76)	1.96 (1.22; 3.13)**	3.93 (2.32; 6.64)***	<0.001	1.30 (1.18; 1.43)***

***p<0.05; **p<0.01; ***p<0.001**

GDF15: Growth differentiation factor 15; **Q:** Quartile; **SPPB:** Short Physical Performance Battery

Model 1: Logistic regression model adjusted for: age, sex, and education.

Model 2: Further adjusted for smoking status, alcohol consumption, physical activity, time watching TV, energy intake, sleep time and diet quality (MEDAS score).

Model 3: Further adjusted for body mass index, systolic blood pressure, serum glucose, serum creatinine, and serum LDL-cholesterol.

eTable 6. Odds ratios (95% confidence interval) for the prospective association of GDF15 with the risk of reduced lower-extremity performance, impaired agility and mobility, weakness and frailty over 2.2 years, excluding participants with cardiovascular disease and/or diabetes at baseline.

	GDF15 (pg/mL)					
	**Q1** (≤ 846)	**Q2** (>846 to ≤ 1086)	**Q3** (>1086 to ≤1422)	**Q4** (> 1422)	**p-trend**	**per 25% increase**
**Reduced lower-extremity performance (SPPB ≤9)**						
No. cases/N (%)	40/292 (13.70%)	49/261 (18.77%)	62/237 (26.16%)	41/135 (30.37%)		192/925 (20.76%)
Model 1	Ref.	1.43 (0.89; 2.29)	1.82 (1.14; 2.91)*	2.23 (1.31; 3.82)**	0.002	1.18 (1.07; 1.30)**
Model 2	Ref.	1.40 (0.86; 2.27)	1.71 (1.06; 2.78)*	2.19 (1.25; 3.82)**	0.004	1.17 (1.05; 1.29)**
Model 3	Ref.	1.38 (0.85; 2.25)	1.73 (1.06; 2.82)*	2.26 (1.25; 4.07)**	0.004	1.18 (1.06; 1.31)**
**Impaired agility**						
No. cases/N (%)	27/338 (7.99%)	33/320 (10.31%)	38/258 (14.73%)	19/145 (13.10%)		117/1061 (11.03%)
Model 1	Ref.	1.24 (0.72; 2.15)	1.70 (0.98; 2.94)	1.44 (0.74; 2.80)	0.113	1.11 (0.98; 1.25)
Model 2	Ref.	1.23 (0.71; 2.15)	1.66 (0.95; 2.91)	1.46 (0.74; 2.88)	0.123	1.10 (0.98; 1.25)
Model 3	Ref.	1.19 (0.67; 2.10)	1.79 (1.00; 3.19)*	1.51 (0.74; 3.09)	0.089	1.13 (0.99; 1.28)
**Impaired mobility**						
No. cases/N (%)	23/369 (6.24%)	13/340 (3.82%)	35/294 (11.90%)	20/169 (11.83%)		91/1172 (7.76%)
Model 1	Ref.	0.58 (0.28; 1.18)	1.80 (1.00; 3.25)	1.89 (0.96; 3.74)	0.007	1.21 (1.06; 1.37)**
Model 2	Ref.	0.56 (0.27; 1.15)	1.75 (0.96; 3.19)	1.96 (0.98; 3.92)	0.007	1.22 (1.07; 1.39)**
Model 3	Ref.	0.55 (0.27; 1.13)	1.76 (0.96; 3.23)	2.06 (1.00; 4.23)*	0.006	1.24 (1.08; 1.43)**
**Weakness**						
No. cases/N (%)	41/325 (12.62%)	39/289 (13.49%)	39/247 (15.79%)	26/132 (19.70%)		145/993 (14.61%)
Model 1	Ref.	1.01 (0.63; 1.64)	1.05 (0.64; 1.73)	1.19 (0.67; 2.12)	0.586	1.04 (0.93; 1.15)
Model 2	Ref.	1.01 (0.62; 1.65)	1.00 (0.60; 1.66)	1.20 (0.66; 2.19)	0.652	1.03 (0.92; 1.15)
Model 3	Ref.	1.07 (0.65; 1.77)	1.08 (0.64; 1.82)	1.43 (0.75; 2.73)	0.358	1.09 (0.96; 1.23)
**Frailty**						
No. cases/N (%)	25/392 (6.38%)	22/355 (6.20%)	28/301 (9.30%)	23/176 (13.07%)		98/1224 (8.00%)
Model 1	Ref.	0.98 (0.53; 1.81)	1.40 (0.76; 2.55)	2.24 (1.16; 4.32)*	0.014	1.19 (1.05; 1.35)**
Model 2	Ref.	0.99 (0.53; 1.86)	1.46 (0.78; 2.73)	2.32 (1.17; 4.62)*	0.013	1.18 (1.04; 1.35)*
Model 3	Ref.	1.00 (0.53; 1.90)	1.60 (0.85; 3.04)	2.55 (1.23; 5.28)*	0.008	1.21 (1.05; 1.40)**

***p<0.05; **p<0.01; ***p<0.001**

GDF15: Growth differentiation factor 15; **Q:** Quartile; **SPPB:** Short Physical Performance Battery

Model 1: Logistic regression model adjusted for: age, sex, and education.

Model 2: Further adjusted for smoking status, alcohol consumption, physical activity, time watching TV, energy intake, sleep time and diet quality (MEDAS score).

Model 3: Further adjusted for body mass index, systolic blood pressure, serum glucose, serum creatinine, and serum LDL-cholesterol.

eTable 7. Beta coefficients (95% confidence interval) for the cross-sectional association of GDF15 with lower-extremity performance, grip strength and Deficit Accumulation Index (DAI), at baseline.

	GDF15 (pg/mL)					
	**Q1** (≤ 877)	**Q2** (>877 to ≤1158)	**Q3** (>1158 to ≤1619)	**Q4** (> 1619)	**p-trend**	**per 25% increase**
**Lower extremity performance (SPPB Global Score)**						
N	619	620	617	619		2475
Model 1	Ref.	0.03 (-0.16; 0.22)	-0.04 (-0.23; 0.16)	-0.49 (-0.69; -0.28)***	<0.001	-0.11 (-0.14; -0.08)***
Model 2	Ref.	0.04 (-0.15; 0.23)	0.03 (-0.17; 0.22)	-0.39 (-0.60; -0.19)***	<0.001	-0.09 (-0.12; -0.06)***
Model 3	Ref.	0.06 (-0.13; 0.25)	0.08 (-0.11; 0.27)	-0.24 (-0.46; -0.02)*	0.078	-0.06 (-0.11; -0.03)***
**Grip Strength (kg)**						
N	618	619	617	617		2471
Model 1	Ref.	-0.21 (-0.86; 0.43)	-0.14 (-0.80; 0.51)	-1.57 (-2.24; -0.90)***	<0.001	-0.33 (-0.44; -0.22)***
Model 2	Ref.	-0.24 (-0.87; 0.40)	-0.06 (-0.71; 0.59)	-1.35 (-2.02; -0.68)***	<0.001	-0.29 (-0.40; -0.18)***
Model 3	Ref.	-0.35 (-0.99; 0.29)	-0.19 (-0.85; 0.46)	-1.60 (-2.34; -0.86)***	<0.001	-0.36 (-0.49; -0.24)***
**DAI score**						
N	621	619	620	619		2479
Model 1	Ref.	0.81 (-0.17; 1.79)	2.04 (1.04; 3.04)***	6.01 (4.99; 7.04)***	<0.001	1.18 (1.02; 1.34)***
Model 2	Ref.	0.68 (-0.25; 1.62)	1.57 (0.62; 2.52)**	5.28 (4.30; 6.27)***	<0.001	1.05 (0.89; 1.20)***
Model 3	Ref.	0.51 (-0.38; 1.40)	1.14 (0.23; 2.05)*	4.22 (3.22; 5.23)***	<0.001	0.76 (0.59; 0.93)***

***p<0.05; **p<0.01; ***p<0.001**

GDF15: Growth differentiation factor 15; **Q**: Quartile; **DAI:** Deficit Accumulation Index; **SPPB:** Short Physical Performance Battery

Model 1: Linear regression model adjusted for: age, sex, and education.

Model 2: Further adjusted for smoking status, alcohol consumption, physical activity, time watching TV, energy intake, sleep time and diet quality (MEDAS score).

Model 3: Further adjusted for body mass index, systolic blood pressure, serum glucose, serum creatinine, serum LDL-cholesterol, and cardiovascular disease and diabetes except for the association with the DAI score.

eTable 8. Beta coefficients (95% confidence interval) for the prospective association of GDF15 with changes in lower-extremity performance, grip strength and Deficit Accumulation Index (DAI), after the 2.2-year follow-up.

	GDF15 (pg/mL)					
	**Q1** (≤877)	**Q2** (>877 to ≤1158)	**Q3** (>1158 to ≤1619)	**Q4** (>1619)	**p-trend**	**per 25% increase**
**Change in the SPPB global score**						
N	394	379	365	338		1476
Model 1	Ref.	-0.00 (-0.24; 0.23)	-0.29 (-0.53; -0.06)*	-0.51 (-0.76; -0.26)***	<0.001	-0.12 (-0.16; -0.08)***
Model 2	Ref.	0.02 (-0.21; 0.25)	-0.23 (-0.47; 0.01)	-0.44 (-0.69; -0.19)***	<0.001	-0.11 (-0.15; -0.06)***
Model 3	Ref.	0.04 (-0.20; 0.27)	-0.21 (-0.44; 0.03)	-0.36 (-0.63; -0.09)*	0.004	-0.10 (-0.15; -0.05)***
**Change in grip strength (kg)**						
N	394	379	365	338		1476
Model 1	Ref.	-0.69 (-1.83; 0.46)	0.88 (-0.29; 2.06)	-0.37 (-1.60; 0.86)	0.780	-0.02 (-0.23; 0.19)
Model 2	Ref.	-0.66 (-1.82; 0.49)	0.92 (-0.26; 2.11)	-0.43 (-1.68; 0.83)	0.829	-0.02 (-0.24; 0.19)
Model 3	Ref.	-0.77 (-1.93; 0.39)	0.77 (-0.43; 1.96)	-0.84 (-2.22; 0.54)	0.828	-0.10 (-0.34; 0.15)
**Change in the DAI score**						
N	454	433	419	385		1691
Model 1	Ref.	0.56 (-0.33; 1.45)	0.65 (-0.26; 1.56)	1.76 (0.78; 2.75)***	0.001	0.30 (0.13; 0.47)***
Model 2	Ref.	0.44 (-0.45; 1.33)	0.54 (-0.37; 1.46)	1.66 (0.66; 2.65)**	0.002	0.28 (0.11; 0.45)**
Model 3	Ref.	0.54 (-0.36; 1.43)	0.56 (-0.36; 1.49)	1.73 (0.68; 2.78)**	0.003	0.26 (0.07; 0.45)**

***p<0.05; **p<0.01; ***p<0.001**

GDF15: Growth differentiation factor 15; **Q**: Quartile; **DAI:** Deficit Accumulation Index; **SPPB:** Short Physical Performance Battery

Model 1: Linear regression model adjusted for: baseline value in the physical function outcome, age, sex, and education.

Model 2: Further adjusted for smoking status, alcohol consumption, physical activity, time watching TV, energy intake, sleep time and diet quality (MEDAS score).

Model 3: Further adjusted for body mass index, systolic blood pressure, serum glucose, serum creatinine, serum LDL-cholesterol, and cardiovascular disease and diabetes except for the association with the DAI score.

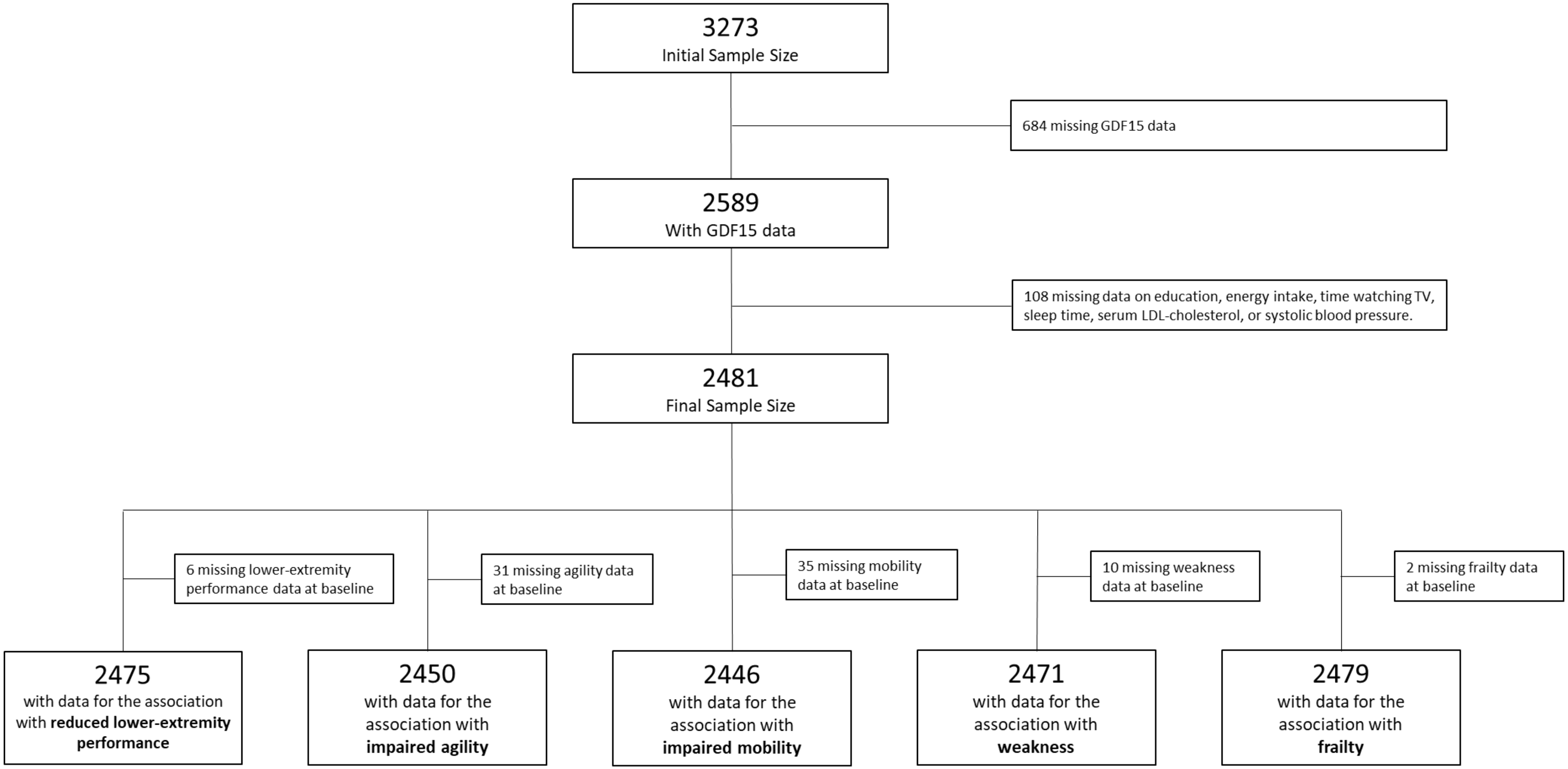

eFigure 1. Participant selection flowchart for cross-sectional analysis.

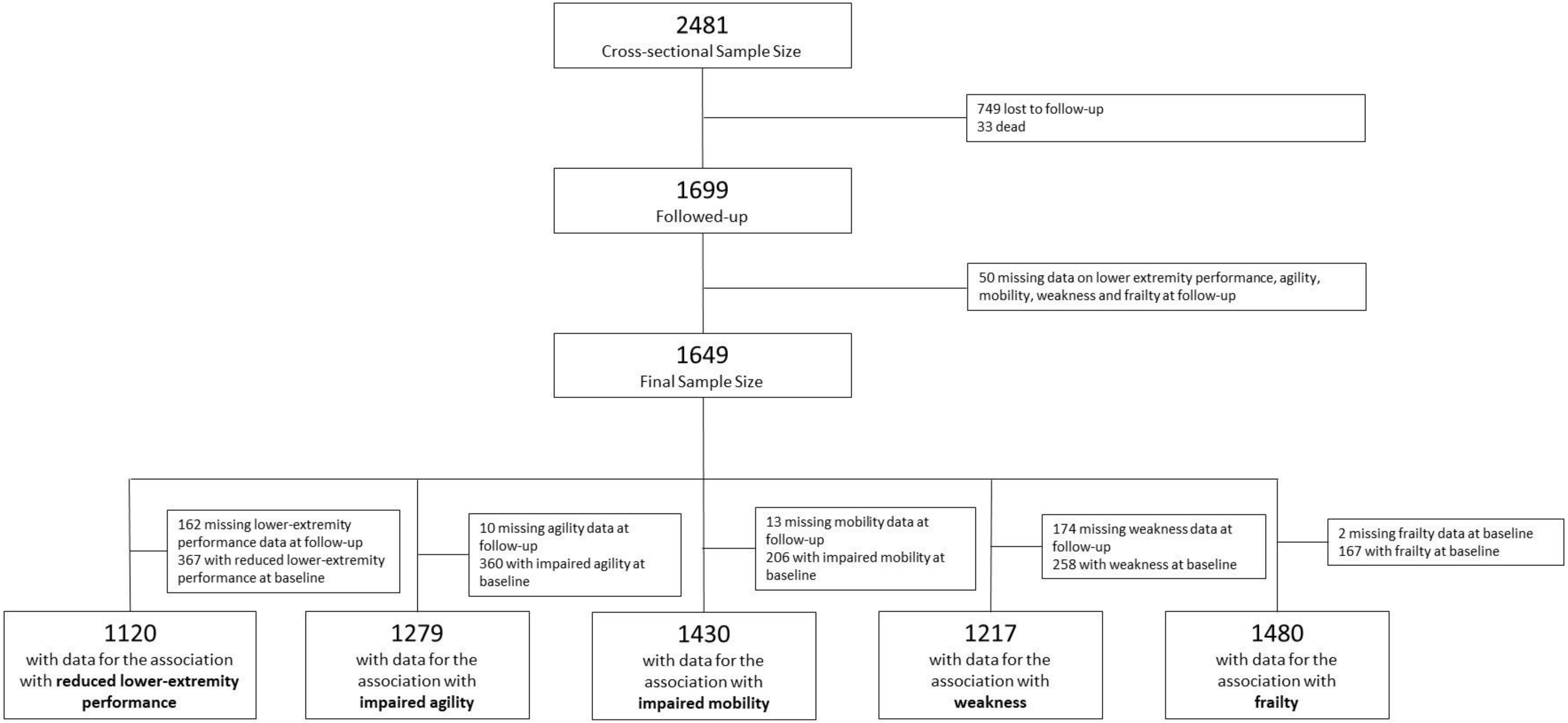

eFigure 2. Participant selection flowchart for prospective analysis.
